# Natural SARS-CoV-2 Infection in Kept Ferrets, Spain

**DOI:** 10.3201/eid2707.210096

**Published:** 2021-07

**Authors:** Christian Gortázar, Sandra Barroso-Arévalo, Elisa Ferreras-Colino, Julio Isla, Gabriela de la Fuente, Belén Rivera, Lucas Domínguez, José de la Fuente, José M. Sánchez-Vizcaíno

**Affiliations:** Instituto de Investigación en Recursos Cinegéticos, Consejo Superior de Investigaciones Científicas, Universidad de Castilla–La Mancha, Ciudad Real, Spain (C. Gortázar, E. Ferreras-Colino, J. de la Fuente);; Universidad Complutense de Madrid, Madrid, Spain (S. Barroso-Arévalo, B. Rivera, L. Domínguez, J.M. Sánchez-Vizcaíno);; Sabiotec, Ciudad Real (J. Isla, G. de la Fuente);; Oklahoma State University, Stillwater, Oklahoma, USA (J. de la Fuente)

**Keywords:** COVID-19, coronavirus disease, SARS-CoV-2, severe acute respiratory syndrome coronavirus 2, viruses, respiratory infections, zoonoses, *mustela putorius furo*, natural infection, pets, work animals, ferrets, Spain

## Abstract

We found severe acute respiratory syndrome coronavirus 2 RNA in 6 (8.4%) of 71 ferrets in central Spain and isolated and sequenced virus from 1 oral and 1 rectal swab specimen. Natural infection occurs in kept ferrets when virus circulation among humans is high. However, small ferret collections probably cannot maintain virus circulation.

Natural infection of animals with severe acute respiratory syndrome coronavirus 2 (SARS-CoV-2) has been reported in pet cats and dogs, zoo felids and apes, and mustelids belonging to the subfamily Mustelinae (*1*). Among mustelids, natural SARS-CoV-2 infections have been recorded in farmed American mink (*Neovison vison*) and sporadically in wild mink (https://promedmail.org/promed-post/?id=8015608) and in a kept pet ferret (*Mustela putorius furo*) from an infected household in Slovenia (https://english.sta.si/2838759/first-case-of-coronavirus-positive-pet-confirmed-in-slovenia). Ferrets are common laboratory models, and experimental infections have evidenced their susceptibility to and ability to transmit the virus to other ferrets. SARS-CoV-2 is shed for up to 8 days postinfection (dpi) in nasal washes, saliva, urine, and feces and is effectively transmitted to naive ferrets by direct contact and through the air (*2*,*3*). Experimentally infected ferrets display either no clinical signs or exhibit elevated body temperature and loss of appetite (*2*,*4*).

Ferrets are common pets (https://www.avma.org/resources-tools/reports-statistics/us-pet-ownership-statistics) and are also used as work animals for rabbit control. However, whether SARS-CoV-2 circulates among kept ferret populations and whether ferrets could contribute to virus maintenance remains unknown.

We studied 71 ferrets belonging to 7 owners; the ferrets were used as working animals for rabbit hunting in Ciudad Real Province, central Spain. All 71 ferrets were included in the study, and none showed clinical signs of any illness. Group sizes ranged from 4 to 21 (mean 10). Twenty ferrets belonging to groups 1 and 2 were resampled 66 days after initial sampling. Information on coronavirus disease in the owners was not available. Sampling took place during August–November 2020. Animal sampling procedures were approved by the Madrid Animal Research Ethics Committee (approval no. CM14/2020). We collected 1 oropharyngeal and 1 rectal swab specimen (DeltaSwab Virus 3 mL; Deltalab, https://www.deltalab.es) from each ferret for RNA extraction.

We detected SARS-CoV-2–specific RNA by using a quantitative reverse transcription PCR assay (qRT-PCR). In brief, we extracted RNA by using the KingFisher Flex System (ThermoFisher, https://www.thermofisher.com). We conducted SARS-CoV-2 RNA detection by using the envelope protein–encoding gene and 2 targets (IP2 and IP4) of the RNA-dependent RNA polymerase gene (*RdRp*) (Appendix Table).

Specimens considered positive by qRT-PCR were subjected to virus isolation in Vero E6 cells. We cultured cells in RPMI-1640 Medium (Sigma Aldrich, https://www.sigmaaldrich.com) supplemented with Gibco 10% fetal bovine serum (ThermoFisher), 100 IU/mL penicillin, and 100 μg/mL streptomycin. We cultured cells in 96-well plates at 37°C with 5% CO_2_ for 24–48 h. We then inoculated cells with 10 μL of the oronasal or fecal swab samples. We used mock-inoculated cells as negative controls. We maintained cultured cells with daily observation of virus-induced cytopathic effect and cellular death. After 6 days, cell cultures were frozen, thawed, and subjected to 3 passages with inoculation of fresh Vero E6 cells with the lysates described. We performed SARS-CoV-2 molecular detection by using qRT-PCR on the supernatants from every passage to confirm presence or absence of the virus in the cell culture.

We obtained sequences from 2 positive samples with 38 primer sets (*5*) and performed sequence analysis by using the Sequencing Analysis 5.3.1 (ThermoFisher), and we used SeqScape 2.5 (ThermoFisher) for sequence assembly, using the SARS-CoV-2 isolate Wuhan-Hu-1 complete genome (GenBank accession no. NC_045512) as reference. We performed phylogenetic analysis by using MEGA X (*6*). We used MUSCLE (*7*) to align spike proteins from the sequences with published sequences.

We found SARS-CoV-2 RNA in swab samples from 6 (8.4%) of the 71 ferrets (Table), belonging to 4 of the 7 groups of ferrets investigated. The likelihood of a swab specimen testing positive was unrelated with age group (<1 vs. >1 year old), sex, and site of sample collection (oral vs. rectal) (p>0.2 by Fisher 2-tailed exact test). We confirmed qRT-PCR results by sequencing the positive PCR product. None of the 20 resampled ferrets was PCR-positive, including 1 ferret that had tested positive 2 months earlier by oropharyngeal swab specimen (cycle threshold 35.38).

**Table:** Kept ferrets testing qRT-PCR–positive for SARS-CoV-2, by sample type, Spain*

Animal ID	Sample type	Lowest C_t_ value†
Ferret 1 G1-H6	Rectal swab	34.5
Ferret 2 G1-H17	Nasal swab	37.29
Ferret 3 G2-H5	Nasal swab	35.38
Ferret 4 G5-H11	Nasal swab	39.83
Ferret 5 G7-H7	Nasal swab	30.59
Ferret 6 G7-H9	Nasal swab	38.91

*C_t_, cycle threshold; qRT-PCR, quantitative reverse-transcription PCR; SARS-CoV-2, severe acute respiratory syndrome coronavirus 2. †A C_t_ cutoff of 40 was used. A result was considered positive for SARS-CoV-2 when the sample showed a positive qRT-PCR result for >2 of the 3 analyzed targets.

We isolated SARS-CoV-2 from the rectal swab specimen of ferret 1 (GenBank accession no. SUB9578702-MZ082987) and the nasal swab specimen of ferret 2 (accession no. SUB9585789-MZ099821) (Table). Phylogenetic analysis showed that spike sequences from ferrets were similar and clustered with a genome from Spain that was included in the alignment (Figure). Ferret sequences had no mutations identified in variants of concern, such as deletions (69–70del and 145del) or mutations (N501Y, A570D, and D614G).

**Figure:**
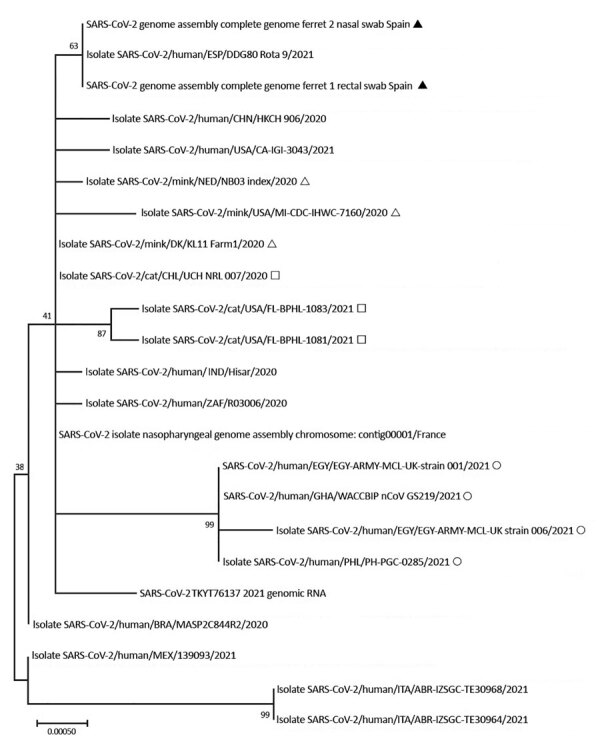
Phylogenetic analysis of SARS-CoV-2 indicating that spike sequences from kept ferrets were similar and clustered with a SARS-CoV-2 genome from Spain that was included in the alignment. Black triangle indicates ferret, open triangle indicates mink; open square indicates cat; open circle indicates human (B1.1.7). Scale bar indicates number of substitutions per site. BRA, Brazil; CHL, Chile; CHN, China; DK, Denmark; EGY, Egypt; ESP, Spain; GHA, Ghana; IND, India; ITA, Italy; MEX, Mexico; NED, Netherlands; PHL, Philippines; SARS-CoV-2, severe acute respiratory syndrome coronavirus 2;TKY, Turkey; UK, United Kingdom; USA, United States; ZAF, South Africa.

We conclude that natural SARS-CoV-2 infection in kept ferrets does occur in circumstances of high viral circulation in the human population (*8*). However, the high cycle thresholds observed and the lack of virus-positive ferrets at resampling suggest that small ferret populations are less able to maintain prolonged virus circulation than large, farmed mink populations (*9*,*10*). Specific guidance on SARS-CoV-2 in ferrets has been made available in the United Kingdom (http://apha.defra.gov.uk/documents/guidance-sars-cov-2-ferrets.pdf) and the United States (https://www.cdc.gov/coronavirus/2019-ncov/animals/pet-store.html).

Appendix Primer sequences and amplified fragment sizes in base pairs used in quantitative reverse-transcription PCR analysis of SARS-CoV-2 in kept ferrets, Spain.
