# Supplementary material for: Natural SARS-CoV-2 Infection in Kept Ferrets, Spain
Source: Emerg Infect Dis. 2021 Jul;27(7):1994–6. doi: 10.3201/eid2707.210096 (PMC8237878; doi:10.3201/eid2707.210096)
Supplement: Appendix — Primer sequences and amplified fragment sizes in base pairs used in quantitative reverse-transcription PCR analysis of SARS-CoV-2 in kept ferrets, Spain. [file 21-0096-Techapp-s1.pdf]

Article DOI: https://doi.org/10.3201/eid2707.210096

Natural SARS-CoV-2 Infection in Kept Ferrets, Spain

Appendix

Appendix Table. Primer sequences and amplified fragment sizes in base pairs*

Primer target	Sequence 5′−3′	PCR fragment size
Gene RdRp/ nCoV_IP2		
nCoV_IP2−12669Fw	ATGAGCTTAGTCCTGTTG	108 bp
nCoV_IP2−12759Rv	CTCCCTTTGTTGTGTTGT	
nCoV_IP2−12696b	AGATGTCTTGTGCTGCCGGTA	
Probe (+)	[5′]Hex [3′]BHQ−1	
Gene RdRp/ nCoV_IP4		
nCoV_IP4−14059Fw	GGTAACTGGTATGATTTCG	107 bp
nCoV_IP4−14146Rv	CTGGTCAAGGTTAATATAGG	
nCoV_IP4−14084	TCATACAAACCACGCCAGG	
Probe(+)	[5′]Fam [3′]BHQ−1	
Gene E/ E_Sarbeco		
E_Sarbeco_F1	ACAGGTACGTTAATAGTTAATAGCGT	125 bp
E_Sarbeco_R2	ATATTGCAGCAGTACGCACACA	
E_Sarbeco_P1	ACACTAGCCATCCTTACTGCGCTTCG	
Probe(+)	[5′]Fam [3′]BHQ−1	

*The qRT-PCR was carried out using the SuperScript III Platinum One-Step qRT-PCR kit (ThermoFisher, https://www.thermofisher.com), according to the manufacturer’s protocol on a CFX Connect Real-Time PCR Detection System (BioRad, https://www.bio-rad.com). The positive control for real-time qRT-PCR was an in vitro transcribed RNA derived from the strain BetaCoV_Wuhan_WIV04_2019 (EPI_ISL_402124), loaned by the Pasteur Institute (Paris, France). Nuclease-free water was used as blank.
